# Biogeography and evolutionary diversification in one of the most widely distributed and species rich genera of the Pacific

**DOI:** 10.1093/aobpla/plw043

**Published:** 2016-08-02

**Authors:** Jason T. Cantley, Adrienne S. Markey, Nathan G. Swenson, Sterling C. Keeley

**Affiliations:** 1 Department of Botany, University of Hawai‘i at Mānoa, Honolulu, HI 96822, USA; 2 Department of Biology, Bucknell University, Lewisburg, PA 17837, USA; 3 Department of Botany, University of Otago, Dunedin 9016, New Zealand; 4 Department of Parks and Wildlife Science and Conservation Division, Kensington, Western Australia 6151, Australia; 5 Department of Biology, University of Maryland, College Park, MD 20742, USA

**Keywords:** Gondwana, island biogeography, long-distance dispersal, New Zealand, Oceania, Oligocene Marine Transgression, repeated dispersals, Zealandia

## Abstract

The historical biogeography of many lineages—of both terrestrial and marine ocean habitats—remains poorly investigated even though remote ocean habitat covers approximately 66% of the Earth’s surface. One such lineage with poorly understood biogeographic affinities across vast ocean habitat is the genus *Coprosma* (Rubiaceae) with numerous species, and a widespread and disjunct distribution among the far-flung insular localities of multiple Pacific Islands. Here, the first taxonomically robust phylogeny for *Coprosma s.s.* was dated using molecular clock techniques and indicated *Coprosma s.s.* diverged from its sister genus *Nertera* likely during or shortly after the Oligocene Marine Transgression of New Zealand. Diversification of the five major clades identified occurred in New Zealand during the Miocene, which was then followed by multiple independent dispersals from New Zealand to various localities in many directions. The pattern of *Coprosma*’s distribution in the Pacific appears stochastic both temporally and spatially, but evolution of an orange to red fruit colour prior to nearly all inferred dispersals hints at endozoochory by birds. The number of inferred long-distance dispersals of *Coprosma* s.s. (>30), and number of repeated dispersals to the same insular locality from unrelated *Coprosma s.s.* sublineages (>8) is perhaps the most currently known for a remote Pacific-centred genus investigated to date. A New Zealand origin for a Pacific-wide dispersal of taxa is not novel, but the manner in which the temporal and spatial distribution for *Coprosma s.s.* was achieved contributes to a novel understanding of the historical biogeography of widespread Pacific genera that have origins in the Southern Hemisphere.

## Abstract (precis)

The largest natural feature on Earth is the Pacific Ocean, which covers over one-third of our planet's surface. This study reconstructed the previously unknown historical biogeography of *Coprosma* (Rubiaceae), which is one of the largest (>110 species) and most widespread flowering plant genera distributed across the Pacific. A New Zealand origin of *Coprosma* was inferred at approximately 25 million years ago (Ma), but most of the distribution was achieved 6 Ma likely by frugivorous birds. Over 30 dispersal events are inferred and >8 locations were colonized more than once, which is perhaps more than any Pacific-centered genus investigated to date.

## Introduction

Biogeographic patterns of plant lineages distributed across extremely remote insular localities remain poorly understudied compared to their mainland counterparts, but are being slowly elucidated from recent studies (Winkworth *et al.* 2002; Gillespie *et al.* 2008; Muellner *et al.* 2008). Perhaps this is a consequence of the difficulties of working among the most isolated localities on Earth, resulting in a poor understanding of species relationships to each other and their area of origin (Price and Wagner 2004; Keeley and Funk 2011). However, a strong rationale for conducting biogeographic and evolutionary focused research on remote islands was recognized as early as the 19^th^ century by Hillebrand (1888, p. xxix) who noted, “the evolution theory could hardly find a more favourable field for observation than an isolated island-group in mid-ocean.” Island floras represent simple systems, in comparison to continental floras, in which specific evolutionary factors of a lineage can be more easily isolated for focused research (Carlquist 1967). It is also possible to deduce a wider understanding of important evolutionary responses by examining common biogeographic patterns of lineages across the oceans and how they are influenced by their biological constraints (i.e. dispersal abilities, niche requirements) within a context of known island geological history (Price and Wagner 2004).

However, a long-standing argument spanning decades, which has perhaps stifled the advancement of island biogeography theory as a whole was the debate between scientists who vehemently supported either vicariance or long-distance dispersal as the process shaping entire floras, to the exclusion of other alternatives (Cowie and Holland 2006; Nelson 2006). Scientists that supported vicariance argued that dispersal was unlikely, impossible to model, unimportant, and therefore by extension not interesting. Scientists supporting the dispersal school of thought refuted this arguing dispersal had significant evolutionary implications, particularly for insular localities that were never connected to tectonically active continents. Cowie and Holland (2006) argue that it is long overdue to accept long-distance dispersal as a well-supported biological process for which new research could and will help predict biogeographic distributions of plants, particularly in isolated landmasses for which dispersal is a fundamental process for arriving organisms to new insular localities (and where vicariance has little to do with the establishment of novel lineages).

Within the last decade, many advances in island biogeography have included dispersal as an integral factor in developing biogeographic modelling tools (Whittaker *et al.* 2008; Matzke 2014). This is particularly notable in research that uses DNA to infer historical biogeography through the theoretical development of models that can predict the past distribution (see Whittaker *et al.* 2008; Gillespie *et al.* 2012; Ree and Smith 2008; Ronquist and Sanmartín 2011; Matzke 2014). These models do not aim to remove vicariance as a plausible explanation used to infer an organism’s past distribution, but rather tend to incorporate both vicariance and dispersal parameters into biologically and geologically plausible scenarios useful to develop a comprehensive understanding of a lineage’s present and past distributions.

The biogeographic patterns of *Coprosma* (Rubiaceae) are among those that have remained poorly understood, despite its place as one of the most widespread and species rich genera across the Pacific Ocean (Cantley *et al.* 2014), which covers approximately one-third of the surface of Earth. The >110 taxa of *Coprosma* are located on nearly every major island archipelago of the Pacific, east to west from the Juan Fernández Islands (near Chile) to Borneo and Java, and south to north from the subantarctic islands south of New Zealand to the Hawaiian Islands. The distribution of *Coprosma* includes nearly every high volcanic island archipelago in the Pacific for which suitable habitat exists. Pollen records on Rapa Nui indicate that *Coprosma* even once occupied this exceptionally isolated island before environmental degradation occurred due to human occupation (Azizi and Flenley 2008; Rull *et al.* 2010). The centre of diversity and hypothesized origin of the genus is New Zealand, where more than 55 species occur (Oliver 1935; Gardner 2002a; Cantley *et al.* 2014). A secondary centre of diversity is recognized in the Hawaiian Islands where there are 16 species (Wagner *et al.* 1999; Cantley *et al.* 2016; Wood *et al.* 2016), and possibly in New Guinea where there is a disputed number of taxa (5–15 species: Gardner 2002a; Utteridge 2002). Smaller numbers of species occur in the Marquesas Islands (6 species: Wagner and Lorence 2011), Lord Howe Island (5 species: Papadopoulos *et al.* 2011), and Australia (8 species: Thompson 2010). The remaining occur as one or two taxa within the floras of numerous other Pacific islands. Despite this extensive range of the genus across the Pacific, very few species of *Coprosma* have a widespread distribution across Pacific landmasses. Moreover, a large proportion of taxa are single-island endemics within larger archipelagos. However, in the case of New Zealand, the majority of species often occur in two or three of the four major islands.

Despite the remarkable distribution of *Coprosma* taxa, our current understanding of their biogeographic affinities to one another are limited at best. Previous taxonomy (Oliver 1935) has recognized a major subgeneric taxonomic division of *Coprosma*; subgenus *Coprosma* consisting of species sharing small leaves and solitary flowers and subgenus *Lucidae* having large leaves and clusters of multiple flowers per inflorescence. Oliver (1935) also noted that species in subgenus *Coprosma* had a western Pacific distribution (i.e. New Zealand, Australia, and Malesia plus one Hawaiian taxon), while species in subgenus Lucidae had an ‘eastern distribution’ (New Zealand and remote Pacific Islands minus one Hawaiian taxon). Heads (1996) was the most recent to propose a comprehensive taxonomic synthesis *Coprosma s.l.* (incl. the sinking of *Nertera* and *Leptostigma* to sections of subgenus *Coprosma*), which included a vicariance based biogeographic hypothesis to explain the observed extant distribution of the genus. Classification therein was based on observed morphological variation across the current distribution of *Coprosma s.l.* taxa for landmasses belonging to the former Gondwana supercontinent. Heads’ vicariance hypothesis invoked the occurrence of a widespread *Coprosma s.l*. ancestral taxon during the Jurassic, which became separated following the rifting apart of Gondwana and now accounts for the east to west biogeographic division of two major subgenera, with significant overlap occurring in New Zealand and minor overlap in the Hawaiian Islands. Gardner (2002b) alternatively suggested that the ‘east-west’ distribution of *Coprosma* could be explained as achieved by dispersal of taxa pre-adapted to various habitats/environmental conditions to similar habitats in new locations within a much shorter time frame (within the Tertiary). More precisely, Gardner suggested that the distribution of small leaved subgenus *Coprosma* taxa to the ‘west’ could be explained by the availability of suitable colder/high elevation habitat such as occurs in the highlands of New Guinea, or in Tasmania. Almost no cold/high elevation habitat exists in the ‘east’ except in the Hawaiian Islands, where a taxon from subgenus *Coprosma* is indeed found. Beyond these subgeneric hypotheses, almost no inferences have been made regarding biogeographic affinities among all remote Pacific taxa apart from loosely grouping some taxa into sections or informal groups (Heads 1996; Oliver 1935).

**Figure 1.**
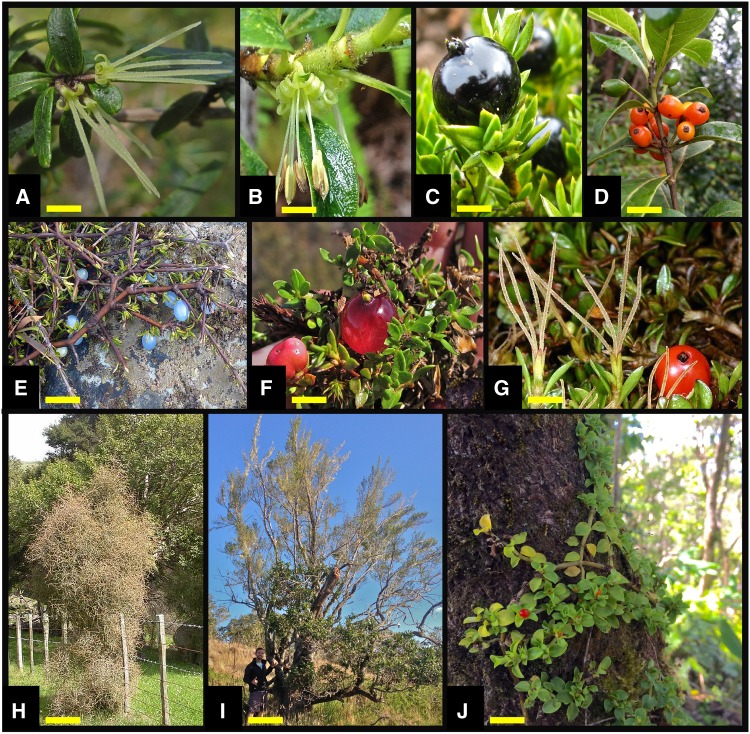
Morphology of *Coprosma* (a-i) and *Nertera* (j). (a) Female flowers of *C. colensoi* (New Zealand), (b) male flowers of *C. ochracea* (Hawai‘i); Fruits of (c) *C. ernodeoides* (Hawai‘i), (d) *C. waimeae* (Hawai‘i), (e) *C. acerosa* (New Zealand), (f) *C. pumila* (Tasmania); (g) Fruits and female flowers of *C. perpusilla* (New Zealand). (h) *C. virescens* showing divaricating shrub habit (New Zealand), (i) *C. montana* of subalpine shrublands (Hawai‘i); (j) Bisexual and herbaceous sister genus taxon *Nertera granadensis*. Photos a & f by AM; b-d & i-j by JTC; e & g-h **©** David Glenny and Jane Cruikshank of Landcare Research; used with permission. Yellow scale bars: a= 7.5 mm; b,c,f,g= 5 mm; e= 20 mm; h= 0.5 m; i= 1 m; j= 10 mm.

The extant distribution of *Coprosma* is highly disjunct as many taxa are separated by thousands of kilometres of open-ocean on islands that were never connected to other landmasses. Seed dispersal in *Coprosma* is usually endozoochorous, where colourful, fleshy fruits (Fig. 1) are taken by vertebrates, including volant birds, which may account for movement over water (see Oliver 1935; Allan 1961; Carlquist 1967). Long-distance dispersal was almost certainly a key in achieving the extant distribution observed in *Coprosma*, but vicariance could also have played a role. For example, *Coprosma* taxa in New Zealand, Australia, and Malesia could represent relics on ancient geologies from a formerly contiguous distribution of a widespread ancestor from when Gondwana was a connected landmass. But, most island localities with *Coprosma* are volcanic in origin, young in age, and have developed *de novo* from the ocean floor. These localities were never connected to any continental landmass (see Price and Wagner 2004), which excludes vicariance as a plausible explanation for the occurrence of *Coprosma* in these localities. Therefore, the primary biogeographic hypothesis of vicariance driving the extant distribution of *Coprosma* after Heads (1996) is flawed, at least in part when considering remote Pacific Islands distributions. Therefore, the long-distance dispersal hypothesis after Gardner (2002b) may be more logical for these taxa. Given the complex geologic history of Oceanic landmasses, the unresolved phylogenetic relationships of *Coprosma* taxa, and the unknown age of the genus, it has not been possible to test the specifics of these competing (but not mutually exclusive) biogeographic hypotheses without dated molecular phylogenetic reconstructions and robust taxonomic representation.

It was the purpose of this study to establish relationships among *Coprosma* taxa through the construction of molecular phylogenies in order to gain an understanding of its Pacific-wide biogeography. Molecular clock techniques and ancestral character reconstructions were applied to provide an estimate of locality and character state evolution through time. We specifically aimed to answer the following questions: a) what are the biogeographic relationships among all Pacific species of *Coprosma*? b) is the extant distribution of *Coprosma* due to vicariance, long-distance dispersal and, or a combination of these processes?, and c) how does the biogeographic pattern of *Coprosma* compare to other Pacific lineages?

## Methods

### Sampling

Of >110 *Coprosma s.s.* taxa, 105 were sampled representing the entire extant geographic distribution of the genus, including all but Bougainville I. (*C. bougainvilleensis*), and Java I. (*C. sundana*). Five genera from tribe Anthospermeae (*Durringtonia, Leptostigma, Nertera, Normandia* and *Opercularia*) were used as outgroups (Anderson *et al.* 2001; Markey *et al.* 2004). A complete list of taxa, collection localities, voucher information, and GenBank accession numbers is given in the supplemental table, [**see Supporting Information—File 1**].

### DNA extraction, amplification and sequencing

DNA was sourced from herbarium collections, field-collected silica dried material, and from the Hawaiian Plant DNA Library (Randell and Morden 1999). Samples were prepared by hand grinding leaves with liquid nitrogen in a mortar and pestle followed by extraction using the DNeasy Plant Mini Kit (Qiagen, Valencia, California, USA). For samples that were difficult to extract DNA, the QiaAmp DNA Stool Mini Kit was used following the manufacturer’s protocols, with a lengthened initial incubation period of 2 h at 70˚C. The primer sequences used and the polymerase chain reaction (PCR) protocols are the same regions (nuclear internal transcribed spacer, external transcribed spacer & chloroplast rps16 intron) by Cantley *et al.* (2014), plus the newly added chloroplast trnQ-rps16 intergenic spacer, which was after Shaw *et al.* (2007). All PCR reactions were performed with 25 µl of reaction cocktail containing 12.75 µl of sterilized H_2_O, 2.0 µl of 20 mM dNTPs (Pharmacia) in an equimolar solution, 2.5 µl of 10x PCR reaction Buffer A (Promega), 1.25 µl of 25 mM MgCl_2_, 0.5 µl 10 mg/ml Bovine Serum Albumin (Sigma), 1 µl of 10 mM of each of the two primers, 0.5 µl Biolase Red *Taq* DNA polymerase enzyme (Bioline) and 4 µl of DNA template. The final amount of DNA template and PCR reaction cocktail and *Taq* was adjusted as necessary to generate sufficient PCR products for DNA sequencing. Samples were purified prior to sequencing using an Exo-Sap enzymatic PCR product pre-sequencing protocol (USB) for 45 min.

A final volume of 8.2 µl was used for sequencing reactions consisting of 2.0 µl of sterilized H_2_O, 3.2 µl of 1 mM primer and 3.0 µl of the purified DNA template. Sequencing was conducted at the Advanced Studies of Genomics, Proteomics and Bioinformatics facility at the University of Hawai‘i at Manoa. Sequences were edited using Sequencher 3.1.10 (Gene Codes Corp., Ann Arbor Michigan, USA) and aligned by MUSCLE (Edgar 2004) using default parameters as implemented in MEGA 5 (Tamura *et al.* 2011). Alignments were then manually inspected and adjusted.

### Phylogenetic and morphological analyses

To test for congruence among gene regions and between chloroplast and nuclear genomes, all gene regions were subjected to the partition homogeneity test (ILD) as implemented in PAUP* ver. 4.0b10 (Swofford 2003) using 1000 replicates, with TBR (tree-bisection reconnection) branch swapping and the MulTrees option turned on. No significant incongruities were found among any of the individual partitions or between the chloroplast genome regions (rps16 intron and trnQ-rps16 intergenic spacer) when compared to nuclear (ITS and ETS) genome regions (*P* = 0.60), so a concatenated data set was used for Maximum Likelihood (ML) and Bayesian Inference (BI) analyses. Prior to running ML and BI analyses, the same best-fit model (GTR + I + Γ) was selected for all individual gene regions as well as the combined nuclear data set using the Akaike Information Criterion (AIC) in jModelTest 2 (Posada and Buckley 2004). ML analyses were implemented in RaxML v7.0.4 (Stamatakis 2006) for individual and combined datasets. Nonparametric bootstrap replicates (1000) for all ML analyses were calculated with the thorough bootstrap replicate option selected and allowing all free model parameters to be estimated.

The BI analyses were implemented in MrBayes 3.1.2 (Hueselenbeck and Ronquist 2001) for individual regions and the concatenated datasets. The combined dataset was divided into six partitions (ITS1, 5.8s, ITS2, ETS, *rps16* intron and *trnQ-rps16*) and Markov Chain Monte Carlo (MCMC) sampling was performed with two replicates of four chains (one hot and three cold) each with a heating temperature of 0.2. Five million generations were completed with sampling every 1000 generations. Burn-in was estimated using Tracer v1.5 (Rambaut and Drummond 2007) with visual inspection of the plotted log-likelihood values. Trees generated before convergence was reached were discarded. The remaining trees were combined into a consensus tree with Bayesian posterior probabilities (PP) calculated for internal node support.

### Fruit colour character reconstructions

Ancestral character state reconstructions were completed for fruit colour of the *Coprosma* species represented in the phylogenetic reconstructions. Fruit colour was coded based on the observed external appearance of a species fruit colour at maturity regardless of whether internal fruit flesh was coloured or translucent. Colours were divided into the following unordered character states: orange (to red), deep violet, black, blue, yellow, polymorphic, non-fleshy fruit, and clear (unpigmented) fruit. For clarity purposes and because many taxa have fruits that range in colour from orange to red, the colour red was included into the orange fruit category. Utilizing Mesquite 2.74 (Maddison and Maddison 2011), fruit colour was traced on the phylogenetic reconstructions using default Maximum Parsimony settings.

### Molecular clock analyses

Prior to implementing the dating analyses in BEAST 1.7.5 (Drummond *et al.* 2012), the best-fit model of molecular evolution was determined for each of the six partitions in jModelTest (Posada and Crandall 1998) using the Bayesian Information Criterion (BIC). The GTR + G model was the selected model for most of the partitions. Alternate models were selected for trnQ-rps16 and ETS; however GTR + G also received a high BIC value. As GTR + G is used natively within BEAST, these models were chosen. Analyses were then run on the CIPRES Science Gateway version 3.2 portal (Miller *et al.* 2012).

To produce dated phylogenetic reconstructions, three separate analyses were performed in BEAST each with independent constraints. A fourth analysis was completed as a total evidence approach in which all constraints were applied to the analysis concurrently. The first analysis constrained the root height of the entire phylogenetic reconstruction between 0 and 50 Ma, as this is a reasonable time estimation of evolution based on previous studies of the Rubiaceae (Bremer and Eriksson 2009). The second analysis included the 0-50 Ma root height and an additional fossil pollen calibration point for the monophyletic lineage of *Coprosma* and *Nertera*. This node was constrained to 5.3-31 Ma with fossil pollen evidence in New Zealand (Graham 2009; MacPhail 1998). The third constraint independently assessed the ITS rate of molecular evolution and the rate range was set to 0.38 x 10 ^−^ ^9^ to 8.34 x 10 ^−^ ^9^ with an initial starting value of 1.99 x 10 ^−^ ^9^ after Kay *et al.* (2006). This range represents estimated ITS rates known for most angiosperms and the initial start value was chosen as the rate found for *Gaertnera* (Rubiaceae), as the most reasonable estimate given the caveat of Kay *et al.* (2006) that phylogenetic relatedness is not necessarily correlated with the rate of molecular change

For all dated phylogenetic reconstructions, the complete concatenated dataset (four DNA regions; 112 taxa) was used. All species of the genus *Coprosma* and *Nertera* (except *C. talbrockiei* and *C. moorei*) were set as monophyletic as indicated from BI and ML analyses. A starting tree was generated without date or rate prior information for ten million generations, which circumvented initial trees starting with likelihoods of negative infinity. Each analysis was run for 300 million generations with sampling occurring every 10 000 generations. Sampled generations were then down-sampled in LogCombiner 1.7.5 (Drummond *et al.* 2012) and a consensus tree was generated using TreeAnnotator 1.7.5 (Drummond *et al.* 2012) with maximum clade credibility, mean node heights and discarded burn in. Trees were visualized in FigTree 1.3.1 (Rambaut and Drummond 2009).

## Results

### Phylogenetic analyses

**Figure 2.**
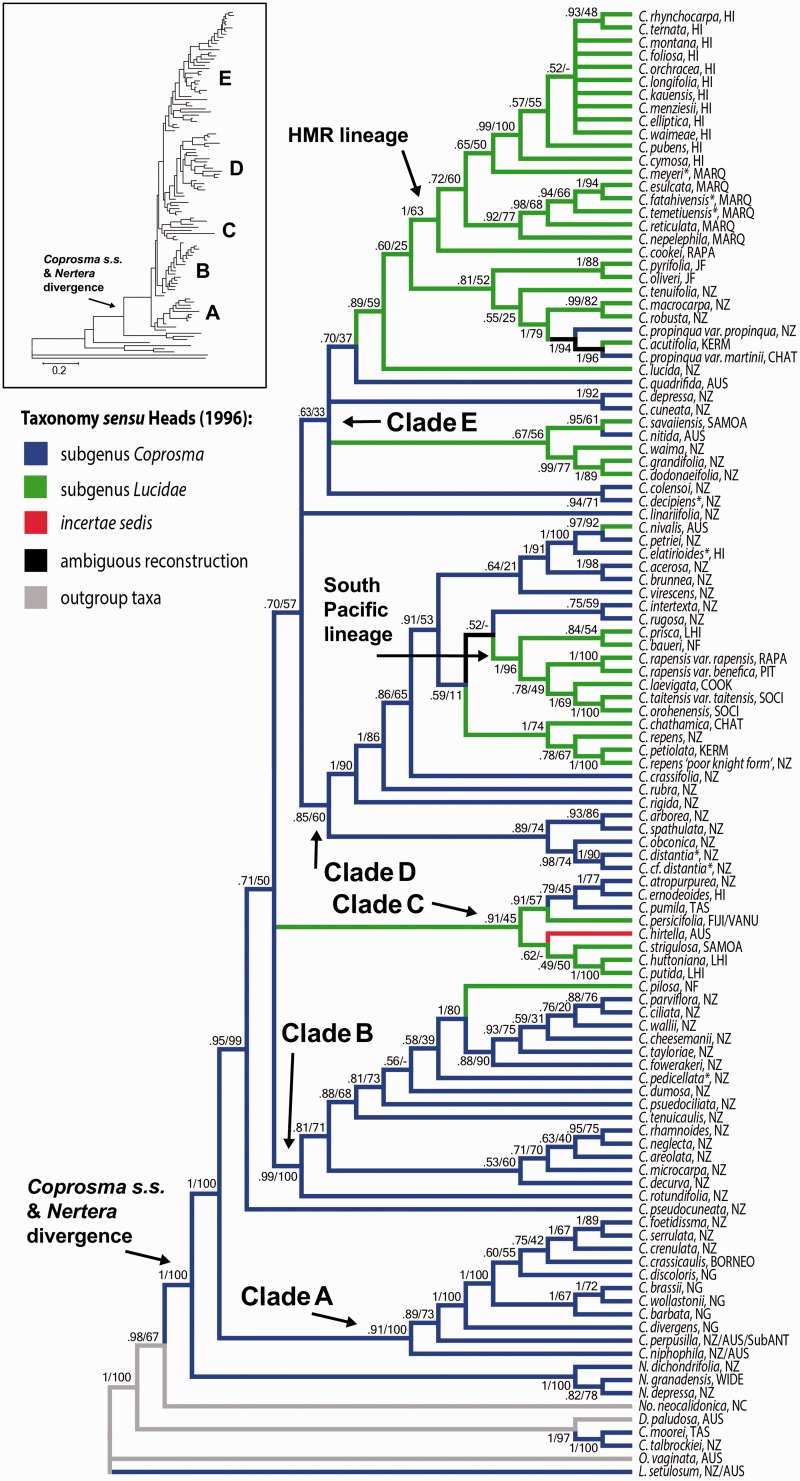
Phylogenetic reconstruction of Bayesian Inference showing a 50% consensus topology based on two nuclear (ITS and ETS) and two plastid (rps16 and trnQ) DNA regions. Numbers above branches represent Bayesian posterior probabilities and Maximum Likelihood bootstrap values, respectively. Branch colours represent taxonomy after Heads (1996) classification. *= taxa described after Heads (1996) subgeneric classification, but for which taxonomic descriptions would ally into Heads’ subgenera. Inset = phylogram of the Bayesian Inference.

The BI and ML phylogenetic analyses agreed in topology and inferred that *Coprosma s.l.* is not monophyletic as *C. talbrockiei* and *C. moorei* are more closely related to *Durringtonia paludosa* than to other *Coprosma* taxa *sensu stricto* (Fig. 2). Furthermore, *Nertera* and *Leptostigma* are nested outside the *Coprosma s.s.* lineage. The phylogenetic analyses did not support the two subgenera *Coprosma* and *Lucidae* of Heads (1996). Alternatively, the analyses suggest that both subgenera are polyphyletic, where subgenus *Lucidae* independently develops from subgenus *Coprosma* (and vice versa) many times. Moreover, *Coprosma s.s.* (i.e. *Coprosma* without the inclusion of *C. talbrockiei* and *C. moorei*), was strongly supported in both analyses with *Nertera* as the sister genus. The two genera are separated by branch lengths that are far longer than the branch lengths found among all *Coprosma s.s.* sublineages (Fig. 2, inset).

Within *Coprosma s.s.*, five well-developed lineages and two monophyletic lineages were recovered (Fig. 2, clades A-E, *C. pseudocuneata*, and *C. linariifolia*). Taxa of Clade A are positioned on a grade between *Nertera* and all other *Coprosma s.s.* taxa. Clade A is composed of eleven taxa from New Zealand (3), New Guinea (5), Borneo (1), plus two taxa that are found in both New Zealand and Australia. Sister to Clade A is the monophyletic lineage of *C. pseudocuneata* plus a polytomy of Clade B, Clade C, and (Clade D + Clade E + *C. linariifolia*). Clade B has 16 New Zealand taxa and *C. pilosa* of Norfolk Island. Clade C of eight taxa, represents six locations (Australia, Fiji, the Hawaiian Islands, New Zealand, Lord Howe Island, and Samoa). The 27 taxa of Clade D are primarily from New Zealand (17), but are also from the Austral Islands (1), Australia (1), Chatham Islands (1), Cook Islands (1), Kermadec Islands (1), Lord Howe Island (1), Norfolk Islands (1), Society Islands (2), and Pitcairn Island (1). Clade E is the largest clade containing 38 taxa and received only marginal support from the phylogenetic analyses. Within Clade E, 12 taxa are from the Hawaiian Islands, 12 are from New Zealand, and six are from the Marquesas Islands. The remaining Clade E taxa are from Australia (2), Chatham Islands (1), Austral Islands (1), Juan Fernandez Islands (2), Kermadec Islands (1), and Samoa (1).

### Molecular clock and distribution of *Coprosma s.s.* taxa

**Figure 3.**
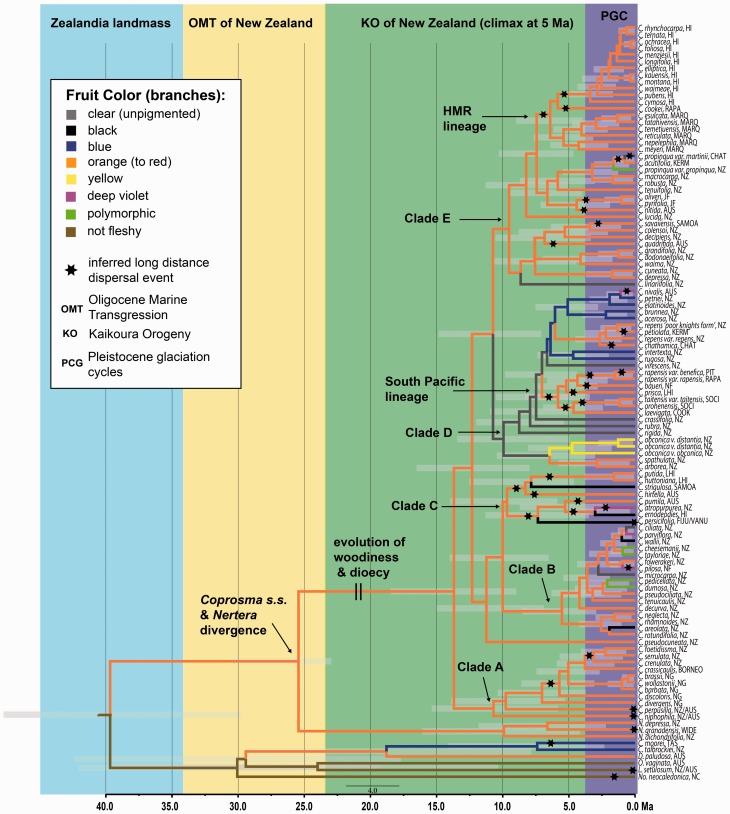
Chronogram from molecular clock analyses. Branch node positions indicate mean prior distributions and light gray bar spanning nodes are the estimated 95% HPD ranges. Branch colours indicate inferred fruit colour inheritance. Colour blocks behind cladogram indicate relevant periods of paleoclimate in New Zealand discussed further in the text.

The three BEAST permutations for dating the genus with a molecular clock (root height constraint, *Coprosma *+* Nertera* fossil constraint, and combined root height and *Coprosma *+* Nertera* fossil constraint) all inferred similar age estimates at nodes. However, the 95% HPD confidence intervals were slightly smaller in the combined analyses (i.e. the total evidence approach) and are presented here (Fig. 3). There are some minor topological differences between the molecular clock analyses and the phylogenetic analyses due to the requirement of full bifurcation within BEAST analyses. Namely, the position of Clade C, the two monotypic lineages, *C. linariifolia* and *C. pseudocuneata*, are all nested one step in their phylogenetic positions. Also, within Clade E the Hawaiian, Marquesan, and *C. cookei* from Rapa resolve differently, but without sufficient posterior probability support.

Clocking analyses infer that *Coprosma s.s.* diverged from *Nertera* around 25 Ma during the Oligocene. The ancestral state reconstruction analyses indicated that the most recent common ancestors (MRCA) for both *Coprosma s.s.* and *Coprosma s.s.* + *Nertera* was New Zealand. The divergences establishing the major extant lineages (Clades A–E) occurred during the mid Miocene, roughly 10 Ma. Furthermore, New Zealand was inferred as their ancestral geographic origin. Diversification within all clades was predominantly initiated around 7 Ma.

*Coprosma s.s.* taxa that are not from New Zealand are found in every clade. Within Clade A, all Malesian (New Guinea, Indonesia, and Borneo) taxa form a monophyletic lineage if three nested New Zealand taxa (*C. crenulata, C. foetidissima*, and *C. serrulata*) are considered. Divergence commenced among Malesia taxa about 7 Ma and the nested New Zealand taxa diverged from Malesian taxa approximately 4 Ma. Of the 18 taxa of Clade B, 17 are from New Zealand and one occurs in Norfolk Island. Clade B is the most recently derived lineage as it is inferred most divergence occurred from the Pliocene/late Miocene (6 Ma) onward. The eight taxa of Clade C represent six locations. An initial divergence within Clade C separates taxa of Fiji, the Hawaiian Islands, Tasmania and New Zealand from taxa from Lord Howe Island, Samoa and Australia. Because of this, it was not possible to infer the location for its MRCA, but the MRCA of Clade C + Clade B is inferred as New Zealand. The MRCA of Clade D is inferred as occurring in New Zealand, but two nested lineages off taxa occur elsewhere: a) a lineage including *C. petiolata* of the Kermadec Islands and *C. chathamica* of the Chatham Islands, and b) a radiation of seven taxa from the Austral Islands, Cook Islands, Lord Howe Island, Norfolk Island, Pitcairn Island, Society Islands (South Pacific lineage). One Australian taxon, *C. nivalis*, is also nested in Clade D. Clade E is the largest lineage with 38 taxa, and includes four lineages with Pacific taxa and three independent Australian lineages. Three of the Pacific lineages are minor: two Juan Fernández Island taxa (*C. oliveri* and *C. pyrifolia*), a Samoan (*C. savaiiensis*) taxon, and a Kermadec Island (*C. acutifolia*) taxon. The fourth Pacific lineage of Clade E is the largest radiation outside of New Zealand, which includes taxa from the Hawaiian Islands, the Marquesas Islands, and Rapa Iti of the Austral Islands (HMR lineage) with a divergence indicated during the late Miocene.

### Inferred dispersals and fruit colour correlation

There were 30 long distance dispersal events inferred for *Coprosma s.s.* (Fig. 3). Dispersal events were inferred from known information regarding the historical position of islands and continental landmasses. Given the age estimates of *Coprosma s.s.*, all geographic disjunctions were considered long-distance dispersal events as these distant locations were never connected during the time of *Coprosma s.s.* evolution. Therefore, a vicariance explanation for all taxa of the genus would not have been possible as suggested by Heads (1996). However, localized vicariance or allopatric speciation was not ruled out from our analyses for recent radiations (i.e. closely related taxa of New Zealand in Clade B, New Guinea taxa of Clade A, or some Hawaiian taxa of Clade E).

An orange (to red) fruit colour was inferred for the MRCA of *Coprosma s.s.* taxa (Fig. 3). Fruit colours other than orange are largely confined to New Zealand, with the exception of two black fruited taxa (Fiji/Vanuatu, Hawai‘i) and two violet-coloured taxa (Norfolk Is., Australia). Orange fruit colouration was already developed prior to all but one inferred dispersal event of taxa occurring outside of New Zealand.

## Discussion

### Biogeographic origins of *Coprosma s.s.*

The molecular clock analyses give no support for Heads’ (1996) vicariance hypothesis for *Coprosma* with a Jurassic origin, and do not support an ancient Gondwanan distribution followed by subsequent vicariance to account for the current Pacific distribution of *Coprosma s.s.* Alternatively, the analyses suggest a more recent origin in the late Oligocene, which greatly post-dates the separation of Gondwanan landmasses (125–80 Ma: Lee *et al.* 1988, Mildenhall 1980, McLoughlin 2001, Cooper and Millener 1993, Pole 1994). Heads (1996) largely based his biogeographic hypotheses on morphological groupings of Oliver (1935). However, our analyses do not support this paraphyletic classification as the two subgenera were recovered as highly polyphyletic therefore further weakening the basis for Head’s (1996) vicariance argument.

**Figure 4.**
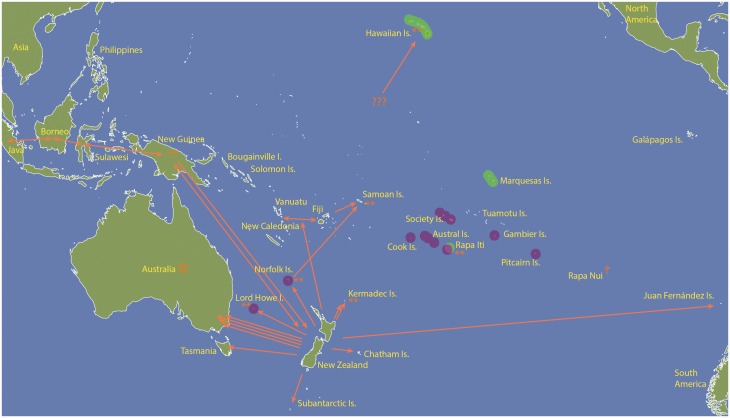
Spatial biogeographic summary of *Coprosma s.s.* Orange arrows indicate inferred directionality of dispersals for *Coprosma s.s.* taxa from phylogenetic analyses, but do not necessarily represent exact dispersal routes. Green and pink polygons indicate the HMR and South Pacific lineages, respectively. Orange asterisks indicate localities for which repeated dispersals were inferred. Orange cross indicates an extinct taxon.

The analyses inferred that *Coprosma s.s.* diverged from *Nertera* during the Oligocene (21–31 Ma 95% HPD) and had New Zealand reconstructed as its site of origin (Figs 3 and 4). The inferred timing of divergence corresponds with a major subduction of the New Zealand landmass ca. 22–25 Ma during the late Oligocene—referred to as the Oligocene Marine Transgression (OMT)—in which at least a significant portion of New Zealand is thought to have become submerged (Stevens *et al.* 1988; Cooper and Millener 1993; Cooper and Cooper 1995). Debate exists around the degree of submergence (i.e. complete or incomplete: Pole 1994; Le Masurier and Landis 1996), and therefore whether older terrestrial lineages could have survived the Oligocene drowning. This argument affects the inference of age on New Zealand’s flora as it is either considered ‘recent of primarily oceanic origins’ or of Gondwana (being relicts of vicariance due to the rifting apart of New Zealand from Australia and Antarctica around 82–85 Ma). The inferred timing of the *Coprosma s.s.* – *Nertera* divergence coincides with the OMT, so it neither supports nor refutes the possibility of a total submergence of New Zealand. It is possible that their most recent common ancestor (MRCA) could have colonized New Zealand from elsewhere following a complete submergence (such as from Australia or Malesia for which extant taxa in both genera are known). Alternatively, their MRCA could have persisted on low-lying emergent islands of New Zealand in an incomplete submergence scenario, albeit likely limited or decreasing in total taxonomic biodiversity due to diminishing habitat remaining on the subsiding islands. An incomplete submergence of New Zealand is suggested by phylogenetic reconstructions of *Onychophora* (Allwood *et al.* 2010) *Astelia* (Birch *et al.* 2012), *Agathis* (Knapp *et al.* 2007) and giant weta taxa (Anostostomatidae: Trewick and Morgan-Richards 2005), but at this point it is not possible to say which is the most accurate scenario regarding the evolution of *Coprosma s.s.* and *Nertera*.

Despite the fact that our analyses indicate the origin of *Coprosma s.s.* was during the Oligocene, caution is needed with this interpretation as Sharma and Wheeler (2013) point out that New Zealand geology predisposes endemic lineages to root age shifts when using molecular clock techniques. In particular, they indicate analyses can infer younger root ages if a mass extinction occurred during the OMT, which could certainly be the case in our investigation of *Coprosma*. Specifically, the 95% HPD bar (31–21 Ma) at the node representing the MRCA of *Coprosma s.s.* and *Nertera* (Fig. 3) extends from the early Oligocene to the early Miocene, effectively spanning the entire timeframe of the OMT. Following the arguments of Sharma and Wheeler (2013), if a taxonomically diverse *Coprosma s.s.* experienced a mass extinction during OMT, then the extinct lineages from older divergences prior to the OMT would not be represented in the clocking analyses, and it is therefore possible that our inferred root age is younger than the actual divergence age. Despite this, the above argument does not support an ancient Gondwanan origin as suggested by Heads (1996). From an independently fossil calibrated analysis on Rubiaceae, Bremer and Eriksson (2009) indicate that divergence within the tribe Anthospermeae (of which *Coprosma* is a member) was no earlier than 47 Ma, well after the split of New Zealand from Australia and Antarctica ca. 82–85 Ma. Furthermore, if the genus were to have originated when Australia, New Zealand, South America, and Antarctica were a combined landmass, one could expect phylogenetic clustering of the extant Australian species, but this is also not the case. All Australian species sampled are not closely related, are recently diverged, and are inferred to be the result of multiple recent long- distance dispersal events from New Zealand (Figs 3 and 4).

### Diversification of New Zealand taxa

Inferred ages of the extant diversity for *Coprosma s.s.* are more reliable than the inferred root age as they are not predisposed to potential age shifts (Sharma and Wheeler 2013). Following the OMT of New Zealand, diversification in all sublineages of *Coprosma s.s.* did not begin until the mid-Miocene (∼16 Ma). All major lineages (i.e. Clades A–E, Fig. 3) were established by the end of the Miocene, and their development is correlated temporally with a rapid rise in elevation and an increase of tectonic activity of New Zealand, known as the Kaikoura Orogeny. The Kaikoura Orogeny commenced as a slow uplift ca. 25 Ma with the activation of the modern Pacific-Australian plate boundary that created the Alpine Fault (Cooper and Cooper 1995; Kamp *et al.* 1992). From 25 to 15 Ma most of the land remained underwater, but from about 15 to 5 Ma, roughly the period inferred in which the major lineages of *Coprosma s.s.* were established, tectonic activity between the Pacific and Australian Plates gave rise to the Southern Alps. The resulting rejuvenated landmass of New Zealand was rugged in topography and much larger in area than prior to the OMT (Ollier 1986).

Drastic changes continued to occur in the New Zealand landscape from the Pliocene to the present (5–0 Ma), where a markedly increased tempo of tectonic activity, volcanism (Ollier 1986; Cox and Findlay 1995; Batt *et al.* 2000), and over 20 glaciation cycles are documented (Suggate, 1990; Pillans and Wright 1992). Taylor (1961) suggested that rigorous living conditions of New Zealand during this period could have initiated a ‘burst in speciation’ for *Coprosma* in which divaricating, semi-prostrate and mat forming habits were selected for in this harsh environment. Taylor (1961) further suggested that the very similar morphological characters, which amount to only minute differences among these species was perhaps evidence that these species were recently evolved. This hypothesis of shifts to subalpine area is supported in a few places by our analyses. For example, within Clade B (Fig. 3) a radiation of 13 strongly divaricating taxa of New Zealand are inferred to have evolved during this period and are today often found in subalpine habitats. Mat-forming taxa such as *C. petriei* (Clade D) and *C. atropurpurea* (Clade C) are also recently evolved, but the two trailing taxa *C. perpusilla* and *C. niphophila* of Clade A do not follow this pattern. Alternatively, their divergence is older and their habit may represent a transitional trailing habit shared with the MRCA of woody *Coprosma s.s.* and herbaceous *Nertera* taxa.

The pattern of diversification among closely related New Zealand species is not always clear because of a lack of total phylogenetic resolution, but a number of evolutionary insights can be made. Many sister species pairs exhibit ecological partitioning, but scarcely differ in morphology, such as between coastal sand dune inhabiting *C. acerosa* and *C. brunnea* of braided inland river beds and scree (Clade D). Moreover, these two species are part of a larger adaptive radiation of blue-fruited taxa that exhibit extensive niche partitioning. Conversely, a divergence pattern also includes quite noticeable changes in morphology among closely related taxa. This is exemplified by three taxa, which are the result of diversification following a single dispersal event from New Guinea to New Zealand less than 6 Ma (Clade A, Fig. 3). The niches of these three taxa are distinct as they represent a) an erect, non-divaricate low shrub with large, coriaceous leaves in steep, rocky montane-subalpine shrublands and snow tussock grasslands (*C. serrulata*) b) a sprawling, divaricate, low shrub with small, coriaceous leaves in rocky, montane-subalpine shrublands and tussock grassland (*C. crenulata*), and c) a tall, erect, non-divaricate shrub with soft leaves occurring in the understory of lowland to montane and subalpine forests (*C. foetidissima*). Further studies with robust, population level sampling are required to discern relationships among morphologically similar taxa of recent divergence (ex. Clade B). In turn, this could shed light on how recent glaciation events of New Zealand affected the biodiversity and biogeography of extant taxa.

### Pacific biogeography of *Coprosma s.s*

The biogeographic pattern of *Coprosma s.s.* across the Pacific is not congruent with an east-west vicariant pattern of a widespread ancestor during Gondwanan times as suggested by Heads (1996). Rather, analyses infer that that remote Pacific *Coprosma s.s.* distribution is due to at least 30 long-distance dispersal events, most originating from New Zealand <8 Ma (Fig. 3, black stars on branches), which supports the habitat based dispersal hypothesis put forth by Gardner (2002b). Species of remote Pacific Islands appear to occur in every habitat that is similar to their sister species pair in New Zealand, and therefore may be found to be pre-adapted to the novel environment that they arrived to following dispersal. For example, the HMR lineage of Clade E shares a very similar habitat niche with its closely related New Zealand congeners and a similar case is true for the South Pacific Lineage of Clade D. The South Pacific Lineage shares morphology and habitat with *C. repens* of New Zealand, but a clear sister lineage has diverged into a blue-fruited adaptive radiation in New Zealand of many habitats and various morphological characters.

Due to the lack of internal node support in the phylogenetic analyses within Clades A–E, it was not always possible to infer dispersal directionality. However, the movement of taxa across the Pacific appears to be stochastic and multi-directional. However, the observed stochasticity is not congruent with wind borne dispersal like the small seeds of *Metrosideros* (Carlquist 1967, Wright *et al.* 2001) or oceanic drift of propagules as in *Pandanus* (Gallaher *et al.* 2015). Alternatively, the pattern matches more closely with other fleshy-fruited genera with remote Pacific distributions like *Astelia* (Birch and Keeley 2013). In all but four cases, fruits of *Coprosma s.s.* taxa found outside New Zealand are orange, like that of many *Astelia* species, and it appears that the orange fruit colour was evolved prior to dispersal to new insular locations (Fig. 3). Thus, bird-mediated dispersal via internal ingestion garners support as a logical hypothesis explaining the extant spatial distribution of *Coprosma s.s.* taxa across the Pacific.

Most dispersals throughout the remote Pacific resulted in only one or two endemic taxa evolving on an island following dispersal from a proximally nearby island, and can be described as ‘once-off’ dispersal events. For example *C. oliveri* and *C. pyrifolia* from the Juan Fernández Islands are the result of a single long-distance dispersal from New Zealand ca. 0–4.5 Ma and are not related to any extant Pacific taxa elsewhere (Fig. 4). Two large lineages from Clade E and Clade D are exceptions in that a dispersal event led to a large species radiation following establishment outside of New Zealand. These two large radiations indicate *Coprosma s.s.* has occurred in the remote Pacific for as long as 8 Ma. The larger of the two, the Hawai‘i-Marquesas-Rapa Iti (HMR) lineage (Fig. 4: green polygons), consists of 19 taxa in Clade E from three geographic localities: the Hawaiian Islands (12), the Marquesas Islands (6), and Rapa Iti of the Austral Islands (1). The morphological similarities of this lineage were discussed recently by Cantley *et al.* (2014) and the three newly described Hawaiian taxa are also expected to belong here based on affinities derived from morphology (Cantley *et al.* 2016; Wood *et al.* 2016). Taxa from each locality represent monophyletic groups, but resolution was not found within them, which confounds the ability to infer the ancestral area for their MRCA after dispersal from New Zealand. The age of the HMR lineage’s MRCA between 8 and 4 Ma is a time when all three archipelagoes had emergent islands, and therefore each archipelago could have served as the site of origin for another archipelago’s taxa. The Marquesas Islands are roughly estimated to be 6 Ma at their oldest (Clouard and Bonneville, 2005) and the oldest age of the current high islands in the Hawaiian Islands is similar plus many older islands now subsided (Clague *et al.* 2010). The Austral Islands have a complex geologic history, and even though Rapa Iti is aged to around 5.1 Ma, many other nearby islands and seamounts are possibly as old as 27 Ma (Bonneville *et al.* 2002). The relationship of *C. cookei* of Rapa Iti to the Hawaiian taxon *C. longifolia*, (and by extension the later described *C. ternata* and *C. kawaikiniensis*) was suggested by Fosberg (1937), but disregarded as parallel development. Although the specific reasons for his suggestion were not explicitly stated, Fosberg’s drawings of the stipules for *C. cookei* are large and sheathing, like that of *C. longifolia*, a character not known elsewhere in the genus.

The second considerably sized Pacific radiation is the South Pacific lineage of Clade D and is in almost complete allopatry from the HMR lineage of Clade E. Overlap occurs only on Rapa Iti, but otherwise the taxa of the South Pacific lineage are found in the Society Islands, Cook Islands, Norfolk Islands, Lord Howe Island, and Pitcairn Island (Fig. 4: pink polygons). Given that the age (3.5–7 Ma) of this lineage extends to older than the oldest age estimates of most of the islands of which these *Coprosma s.s.* taxa currently occur, it is possible their MRCA existed on other, older, now subsided islands (such as in the Tuamotu Islands or Line Islands). Alternatively, dispersal among all locations occurred more recently from New Zealand (5 Ma onwards). The phylogenetic analyses support close relationships among taxa speculated by Fosberg (1937), yet, dispersal directionality among the archipelagos could not be inferred. Fosberg (1937) hypothesized that one group of taxa were centred around *C. taitensis*, (three varieties occurring in the Society Islands [Tahiti, Mo‘orea, Taha‘a and Raiatea Islands]), *C. velutina* (not sampled) of the Austral Islands (Raivavae and Rurutu Islands), and the later discovered *C. orohenesis* of the Society Islands (Tahiti). A second group of related taxa was centred around *C. rapensis var. typica* (Rapa, Austral Islands), *C. rapensis var. benefica* (Pitcairn Island) and *C. rapensis var. mangarevica* (Mangareva of the Gambier Islands). We were unable to obtain DNA specimens of *C. rapensis var. mangarevica*, which is the only *Coprosma s.s.* taxon on Mangareva Island of the Gambier Islands, but we expect it to be closely related to the other varieties due to shared morphology. Additionally, Fosberg (1937) suggested that *C. laevigata* of the Cook Islands was closely related to varieties of *C. rapensis* and this is supported for in our analyses. The remaining two species of South Pacific lineage (*C. baueri* of Norfolk Island and *C. prisca* of Lord Howe Island) are much more western in distribution than the Polynesian species described above. Their relationship to Polynesian taxa was unexpected. Oliver (1935) allied these two species with *C. repens* of New Zealand and *C. petiolata* of Kermadec Islands because they share glossy, morphologically similar leaves.

Repeated dispersals, i.e. two taxa of unrelated lineages within a genus arriving to the same geographic locality independently, is important to consider when discussing the overall biogeographic pattern of *Coprosma s.s.* Repeated dispersals have occurred for at least eight locations: the Hawaiian Islands, Rapa Iti of the Austral Islands, Lord Howe Island, Norfolk Islands, Samoa, Kermadec Islands, Australia, and New Zealand (Fig. 4). It is possible a ninth locality is represented by New Guinea, but we were unable to obtain material of the morphologically distinct *C. archboldiana* from the New Guinean Highlands, which shares morphological affinities with taxa of Clade C rather than to other New Guinea species (Gardner 2002a). At least four independent repeated dispersal events are inferred to mainland Australia. The pattern of repeated dispersals is not novel to *Coprosma s.s.*, but it is perhaps the genus with the most numerous repeated dispersal cases (8 or 9) documented for any Pacific-centred/Oceanic angiosperm genus. Repeated dispersals are known for *Scaevola* (Goodeniaceae) in which three known colonizations to the Hawaiian Islands documented arriving via avian endozoochory and oceanic drift dispersal (Howarth *et al.* 2003; Howarth and Baum 2005). Two colonizations of the Marquesas Islands are inferred for *Melicope* (Rutaceae) (Harbaugh 2008), and two colonizations *Metrosideros* (Myrtaceae) to Lord Howe Island (Papadopulos *et al.* 2011). Currently, only *Santalum* (Santalaceae) compares with *Coprosma s.s.* in total localities for which taxa have repeatedly colonized locations. For *Santalum*, this occurred to three locations: the Bonin Islands, Hawaiian Islands, and Cook Islands (Harbaugh *et al.* 2009).

## Conclusions

The genus *Coprosma s.s.* has a unique biogeographic history among Pacific centred angiosperm genera that was primarily influenced by long-distance dispersal. Assessing the overall pattern of movement inferred for *Coprosma s.s.* across the Pacific indicates no clear progression in size, age or direction of movement for the lineage. The only shared similarity for most taxa is that perhaps they all originated at some point from New Zealand. The biogeographic pattern seems stochastic in process in which movements have occurred in all directions, at all times, and have either radiated into large numbers of new taxa upon arrival to a new locality, or have not. *Coprosma s.s.* achieved its current distribution through more than 30 dispersal events over a relatively short period, geologically speaking, which was likely aided by dispersal via frugivorous birds. There is no evidence that vicariance ever played a role in speciation, but refined studies could show that vicariance has played a minor role in some sister species pairs (i.e. Maui Nui Islands of the Hawaiian Archipelago; Allopatric separation of populations of New Zealand). The genus shares various similarities with other widespread lineages that have considerable diversity in New Zealand plus the remote Pacific such as *Astelia* (Asteliaceae) and *Metrosideros* (Myrtaceae), but the biogeographic relationships of *Coprosma s.s.* are quite different. *Coprosma* and *Astelia* both share fleshy orange fruits that were likely dispersed via avian endozoochory, both are dioecious, both also derive largely from ancestral lineages of New Zealand, and both have cases of long-distance dispersal events (Birch *et al.* 2012). However, extant taxa of *Astelia* have reached only a small portion of the distribution of *Coprosma* (Birch *et al.* 2012; Birch and Keeley 2013). Conversely, *Metrosideros* has a similarly widespread distribution (Wright *et al.* 2001) as *Coprosma s.s.*, but lacks the level of endemicity and species diversity as *Coprosma s.s.* This could be because of a higher frequency of dispersal of their small wind borne seeds, which maintains a continuance of gene flow from a source population, and thus has slowed the rate of increasing endemicity or for some other unknown difference among the two genera. Within New Zealand, it appears that the extant diversification of *Coprosma s.s.* lineages occurred shortly after the OMT of New Zealand during the Oligocene. Perhaps the diversification of *Coprosma s.s.* at this time indicates that it was among the first of lineages to exploit new heterogeneous habitats of New Zealand that followed the OMT, and resulted in the lineage ranked as the second largest (in terms of number of species of a woody angiosperm lineage (after *Hebe* [Plantaginaceae]) in New Zealand (Wagstaff and Garnock-Jones 1998). This research elucidated the biogeographic history of *Coprosma* for the first time indicating relationships among distant Pacific taxa that were previously unknown. *Coprosma* is one of the most species rich genera of the wider Pacific flora and this research highlights that this diversity and widespread distribution was achieved in a complex manner of a short period of time.

## Sources of Funding

Funding for this study was provided through the American Society of Plant Taxonomists Graduate Student Research Grant, University of Hawai‘i at Mānoa Charles Lamoureux Fellowship in Plant Conservation, the UHM Graduate Student Organization and the Department of Plant Biology at Michigan State University.

## Contributions by the Authors

JTC contributed to the primary data collection and writing, SCK served to greatly develop ideas and data interpretation, AM was integral to the collection of NZ taxa in the field and both AM & NGS contributed to the revision of manuscript drafts.

## Conflict of Interest Statement

None declared.

## Supplementary Material

Supplementary Data

## Supporting Information

The following additional information is available in the online version of this article —

 **File 1.** Table. Voucher information and GenBank accession numbers for taxa used in this study. C. = *Coprosma*; D. = *Durringtonia*; N. = *Nertera*; No. = *Normandia*.
