# Supplementary material for: Biogeography and evolutionary diversification in one of the most widely distributed and species rich genera of the Pacific
Source: AoB Plants. 2016 Aug 2;8:plw043. doi: 10.1093/aobpla/plw043 (PMC4972462; doi:10.1093/aobpla/plw043)
Supplement: Supplementary Data [file supp_8_plw043_index.html]

Biogeography and evolutionary diversification in one of the most widely distributed and species rich genera of the Pacific — Supplementary Data 

# Biogeography and evolutionary diversification in one of the most widely distributed and species rich genera of the Pacific

## Supplementary Data

files

- Supplementary Data - zip file
