# Supplementary material for: Biogeography and evolutionary diversification in one of the most widely distributed and species rich genera of the Pacific
Source: AoB Plants. 2016 Aug 2;8:plw043. doi: 10.1093/aobpla/plw043 (PMC4972462; doi:10.1093/aobpla/plw043)
Supplement: Supplementary Data [file supp_plw043_suppl_data.zip › aobplants-15339-s01.docx]

| **Supplemental Table 1. Voucher information and GenBank accession numbers. C. = Coprosma; D. = Durringtonia; N. = Nertera; No. = Normandia; O. = Opercularia.** | | | | | | | |
| --- | --- | --- | --- | --- | --- | --- | --- |
| **Species** | **Collector and Collection Number** | **Herbarium** | **Collection Locality** | **ITS Genbank Accession No.** | **ETS Genbank Accession No.** | **rps16 Genbank Accession No.** | **trnQ-rps16 Accession No.** |
| *C. acerosa* | A. Markey & J. Cantley NZ242 | HAW | Dunedin, New Zealand | KF688388 | KF688305 | KF688223 | KT861921 |
| *C. acutifolia* | J. Cantley 227 | HAW | (Kermadec Is.) cult. New Zealand | KF688389 | KF688306 | KF688224 | KT861922 |
| *C. arborea* | J. Cantley 198 | HAW | cult. Auckland, New Zealand | EU169116.1 | GQ130308.1 | KT861908 | - |
| *C. areolata* | A. Markey & J. Cantley NZ241 | HAW | Auckland, New Zealand | KF688390 | KF688307 | KF688225 | KT861923 |
| *C. atropurpurea* | A. Markey & J. Cantley NZ206 | HAW | South Island, New Zealand | KF688424 | KF688340 | KF688256 | KT861953 |
| *C. barbata* | T. Utteridge 34327 | KEW | Mt. Jaya, Indonesia | KF688467 | KF688382 | KF688299 | KT861990 |
| *C. baueri* | R. O. Gardner 5901 | BISH | Anson Bay, Norfolk Is. | KF688464 | KF688379 | KF688296 | KT861987 |
| *C. brunnea* | A. Markey & J. Cantley NZ221 | HAW | Arthur's Pass, New Zealand | KF688391 | KF688308 | KF688226 | KT861924 |
| *C. chathamica* | J. Cantley 219 | HAW | (Chatham Is.) cult. Wellington New Zealand | KF688392 | KF688309 | KF688227 | KT861925 |
| *C. cheesmanii* | A. Markey & J. Cantley NZ215 | HAW | Dunedin, New Zealand | KF688393 | KF688310 | KF688228 | KT861926 |
| *C. ciliata* | J. Cantley s.n. | HAW | cult. Wellington, New Zealand | KF688425 | KF688341 | KF688257 | KT861954 |
| *C. colensoi* | A. Markey & J. Cantley NZ237 | HAW | Catlins NP, New Zealand | AY357777.1 | KT861898 | JF950822.1 | KT861998 |
| *C. cookei* | T.J. Motley s.n. | PTBG | Rapa Iti, Austral Is. | KF688456 | KF688371 | KF688288 | - |
| *C. crassicaulis* | Skottsberg s.n. | GB | Mt. Kinabalu, Borneo | KF688394 | KF688311 | - | - |
| *C. crassifolia* | A. Markey & J. Cantley NZ240 | HAW | New Zealand | KF688395 | KF688312 | KF688229 | KT861927 |
| *C. crenulata* | A. Markey & J. Cantley NZ214 | HAW | Arthur's Pass, New Zealand | KF688396 | KF688313 | KF688230 | KT861928 |
| *C. cuneata* | A. Markey NZ414 | CHR | Blue Mtns, New Zealand | JF950726.1 | KF688356 | JF950826.1 | KT861997 |
| *C. cymosa* | S. Perlman | NTBG | Hawaii, Hawaii | KF688462 | KF688377 | KF688294 | KT861985 |
| *C. decipiens* | J. Smith-Dodsworth s.n. | AK | New Zealand | KF688474 | KT861899 | KT861909 | KT862003 |
| *C. decurva* | A. Markey & J. Cantley NZ222 | HAW | Mt. Cargill, New Zealand | KF688397 | KF688314 | KF688231 | KT861929 |
| *C. depressa* | A. Markey & J. Cantley NZ205 | HAW | New Zealand | KF688475 | KT861900 | KF688303 | KT861996 |
| *C. discolor* | not vouchered | - | New Guinea | KF688398 | KF688315 | - | - |
| *C. divergens* | M. Prebble s.n. | CHR | New Guinea | KF688399 | KF688316 | KF688232 | KT861930 |
| *C. dodonaeifolia* | D. Glenny 10594 | CHR | Auckland, New Zealand | KF688431 | KF688347 | KF688263 | KT861960 |
| *C. dumosa* | A. Markey & J. Cantley NZ234 | HAW | New Zealand | KF688426 | KF688342 | KF688258 | KT861955 |
| *C. elatirioides* | A. Markey & J. Cantley NZ226 | HAW | New Zealand | KF688400 | KF688317 | KF688233 | KT861931 |
| *C. elliptica* | T. Flynn & J. Cantley 7475 | NTBG | Kauai, Hawaii | KF688455 | KF688370 | KF688287 | KT861979 |
| *C. ernodeoides* | T. Gallaher 330 | HAW | Hawaii, Hawaii | KF688436 | KF688351 | KF688268 | KT861965 |
| *C. esulcata* | J-Y. Meyer s.n. | PTBG | Ua Pou, Marquesas | KF688458 | KF688373 | KF688290 | KT861981 |
| *C. fatuhivaensis* | K. Wood 10137 | PTBG | Fatu Hiva, Marquesas | KF688457 | KF688372 | KF688289 | KT861980 |
| *C. foetidissima* | J. Cantley 219 | HAW | Wellington, New Zealand | KF688469 | KF688384 | KF688301 | KT861992 |
| *C. foliosa* | J. Cantley JC-207 | HAW | Oahu, Hawaii | KF688447 | KF688362 | KF688279 | KT861975 |
| *C. fowerakeri* | A. Markey & J. Cantley NZ212 | HAW | Arthur's Pass, New Zealand | KF688401 | KF688318 | KF688234 | KT861932 |
| *C. grandifolia* | J. Cantley JC-221 | HAW | Wellington, New Zealand | KF688402 | KF688319 | KF688235 | KT861933 |
| *C. hirtella* | Thompson & Hewat 883 | BISH | NSW, Australia | JF950730.1 | KT861901 | JF950831.1 | KT862000 |
| *C. huttoniana* | C. Beard s.n. | AK | Lord Howe Is. | JF950734.1 | KT861902 | JF950835.1 | KT862001 |
| *C. inopinata* | Papadopulos s.n. | KEW | Lord Howe Is. | JF950736.1 | - | JF950837.1 | - |
| *C. intertexta* | A. Markey & J. Cantley NZ224 | HAW | Cass, New Zealand | KF688403 | KF688320 | KF688236 | KT861934 |
| *C. kauensis* | S. Perlman 18648 | BISH | Kauai, Hawaii | KF688453 | KF688368 | KF688285 | - |
| *C. laevigata* | G. Mccormack, not vouchered | - | Rarotonga, Cook Islands | KF688476 | - | KT861910 | KT862006 |
| *C. lanceolaris* | D. Mueller-Dumbois 92091015 | BISH | Lord Howe Island | JF950740.1 | - | JF950841.1 | - |
| *C. linariifolia* | A. Markey & J. Cantley NZ249 | HAW | Dunedin, New Zealand | JF950741.1 | GQ130313.1 | JF950842.1 | KT861994 |
| *C. longifolia* | J. Cantley 11-250 | HAW | Oahu, Hawaii | KF688445 | KF688360 | KF688277 | KT861973 |
| *C. lucida* | J. Cantley 203 | HAW | Auckland, New Zealand | KF688404 | KF688321 | KF688237 | KT861935 |
| *C. macrocarpa* | A. Markey & J. Cantley NZ208 | HAW | Dunedin, New Zealand | KF688405 | KF688322 | KF688238 | KT861936 |
| *C. menziesii* | J. Cantley 274 | HAW | Hawaii, Hawaii | KF688406 | KF688323 | KF688239 | KT861937 |
| *C. meyeri* | D. Lorence 19659 | NTBG | Hiva Oa, Marquesas | KF688460 | KF688375 | KF688292 | KT861983 |
| *C. microcarpa* | A. Markey & Cantley NZ203 | HAW | South Island, New Zealand | KF688407 | KF688324 | KF688240 | KT861938 |
| *C. montana* | J. Cantley 271 | HAW | Maui, Hawaii | KF688450 | KF688365 | KF688282 | - |
| *C. moorei* | A. Markey 345 | PERTH | Tasmania, Australia | KT861893 | KT861903 | KT861911 | KT862012 |
| *C. neglecta* | J. Cantley 190 | HAW | Auckland, New Zealand | KF688470 | KF688385 | KF688302 | KT861993 |
| *C. nephelephila* | J. Florence 4342 | PTBG | Nuku Hiva, Marquesas | KF688477 | - | KT861912 | KT862007 |
| *C. niphophila* | A. Markey NZ426 | HAW | Coronet Peak, New Zealand | KF688437 | KF688352 | KF688269 | KT861966 |
| *C. nitida* | Croft 10098 | BISH | Australia | JF950743 | - | KT861913 | KT862009 |
| *C. nivalis* | A. Markey NZ167 | CHR | Australia | KF688408 | KF688325 | KF688241 | - |
| *C. obconica var. distantia* | P.J. de Lange 9763 | CHR | Surville Cliffs, New Zealand | KF688441 | KT861904 | KF688273 | KT861969 |
| *C. obconica var. obconica* | J. Cantley s.n. | HAW | Wellington, New Zealand | KF688409 | KF688326 | KF688242 | KT861939 |
| *C. ochracea* | J. Cantley JC-56 | HAW | Oahu, Hawaii | KF688465 | KF688380 | KF688297 | KT861988 |
| *C. oliveri* | T. Steussy 3456 | WU | Juan Fernandez Islands | KT861894 | KT861905 | KT861914 | KT862004 |
| *C. orohenensis* | Gagne & Mont. s.n. | BISH | Mt. Orohena, Society Is. | KF688463 | KF688378 | KF688295 | KT861986 |
| *C. parvifora* | J. Cantley 180 | HAW | New Zealand | KF688429 | KF688345 | KF688261 | KT861958 |
| *C. pedicellata* | B. Clarkson 1023 | AK | North Island, New Zealand | KF688438 | KF688353 | KF688270 | KT861967 |
| *C. perpusilla* | A. Markey & J. Cantley NZ216 | HAW | Arthur's Pass, New Zealand | JF950746.1 | KT861906 | KT861915 | KT861995 |
| *C. persicifolia* | Rova & Gustavsson 2470 | GB | Fiji | AF257909.1 | - | AF257910.1 | - |
| *C. petiolata* | P.J. de Lange K849 | AK | Raoul Island, Kermadec Is. | KF688439 | KF688354 | KF688271 | KT861968 |
| *C. petriei* | A. Markey & J. Cantley NZ200 | HAW | New Zealand | KF688410 | KF688327 | KF688243 | KT861940 |
| *C. pilosa* | D. Mueller-Dumbois 92091609 | BISH | Norfolk Is. | KF688442 | KF688357 | KF688274 | KT861970 |
| *C. prisca* | D. Mueller-Dumbois 92091602 | BISH | Lord Howe Is. | KF688435 | KF688350 | KF688267 | KT861964 |
| *C. propinqua var. martinii* | A. Markey s.n. | CHR | Chatham Is. | KF688411 | KF688328 | KF688244 | - |
| *C. propinqua var. propinqua* | E. Cameron 15619 | AK | New Zealand | KF688440 | KF688355 | KF688272 | KT861941 |
| *C. pseudociliata* | D. Glenny 10647 | CHR | New Zealand | KF688432 | - | KF688264 | KT861961 |
| *C. pseudocuneata* | A. Markey & J. Cantley NZ217 | HAW | Arthur's Pass, New Zealand | KF688412 | KF688329 | KF688245 | KT861942 |
| *C. pubens* | J. Cantley 297 | HAW | Hawaii, Hawaii | KF688448 | KF688363 | KF688280 | KT861976 |
| *C. pumila* | A. Markey TAS06 | WA | Tasmania, Australia | KF688478 | - | FJ695262.1 | - |
| *C. putida* | D. Mueller-Dumbois 92090730 | BISH | Lord Howe Is. | JF950755.1 | - | JF950857.1 | KT862010 |
| *C. pyrifolia* | M. Pilna s.n. | HAW | (Juan-Fernandez Is.) cult. Chile | KF688443 | KF688358 | KF688275 | KT861971 |
| *C. quadrifida* | Beesley 640 | BISH | NSW, Australia | JF950756.1 | - | JF950858.1 | KT862008 |
| *C. rapensis var. benefica* | G. McCormack 72/17 | CHR | Pitcairn Is. | KF688473 | - | KT861916 | KT862002 |
| *C. rapensis var. rapensis* | J-Y. Meyer & Perlman 1021 | BISH | Rapa Iti, Austral Is. | KF688461 | KF688376 | KF688293 | KT861984 |
| *C. repens* | J. Cantley 110 | HAW | (New Zealand) cult. Hawaii | KF688413 | KF688330 | KF688246 | KT861943 |
| *C. repens Poor Knights Form* | J. Cantley NZ206 | HAW | cult. Auckland, New Zealand | KF688430 | KF688346 | KF688262 | KT861959 |
| *C. reticulata* | Perlman 15067 | BISH | Marquesas | KT861895 | KT861907 | - | - |
| *C. rhamnoides* | A. Markey & J. Cantley NZ224 | HAW | New Zealand | KF688414 | KF688331 | KF688247 | KT861944 |
| *C. rhynchocarpa* | J. Cantley 11-262 | HAW | Hawaii, Hawaii | KF688449 | KF688364 | KF688281 | KT861977 |
| *C. rigida* | A. Markey & J. Cantley NZ235 | HAW | Catlins NP, New Zealand | KF688415 | KF688332 | KF688248 | KT861945 |
| *C. robusta* | J. Cantley 217 | HAW | New Zealand | KF688416 | KF688333 | KF688249 | KT861946 |
| *C. rotundifolia* | A. Markey & J. Cantley NZ228 | HAW | New Zealand | KF688417 | KF688334 | KF688250 | KT861947 |
| *C. rubra* | A. Markey & J. Cantley NZ227 | HAW | New Zealand | KF688418 | KF688335 | KF688251 | KT861948 |
| *C. rugosa* | J. Cantley 213 | HAW | Wellington, New Zealand | KF688419 | KF688336 | KF688252 | KT861949 |
| *C. savaiiensis* | A. Whistler 9999 | HAW | Upolu, Samoa | KF688479 | - | KT861917 | KT862005 |
| *C. serrulata* | A. Markey & J. Cantley NZ213 | HAW | Arthur's Pass, New Zealand | KF688420 | KF688337 | KF688253 | KT861950 |
| *C. spathulata* | D. Glenny 10456 | CHR | North Island, New Zealand | KF688433 | KF688348 | KF688265 | KT861962 |
| *C. strigulosa* | A. Whistler 9899 | HAW | Upolu, Samoa | KF688446 | KF688361 | KF688278 | KT861974 |
| *C. taitensis var. taitensis* | J-Y. Meyer | PAP | Society Is. | KF688444 | KF688359 | KF688276 | KT861972 |
| *C. talbrockiei* | A. Markey 179 | CHR | Gouland Downs, South Island, New Zealand | KT861896 | - | KT861918 | - |
| *C. tayloriae* | A. Markey & J. Cantley NZ236 | HAW | New Zealand | KF688427 | KF688343 | KF688259 | KT861956 |
| *C. temetiuensis* | D. Lorence 8931 | PTBG | Marquesas | KF688459 | KF688374 | KF688291 | KT861982 |
| *C. tenuicaulis* | A. Markey NZ507 | WA | New Zealand | KF688434 | KF688349 | KF688266 | KT861963 |
| *C. tenuifolia* | J. Cantley 220 | HAW | New Zealand | KF688428 | KF688344 | KF688260 | KT861957 |
| *C. ternata* | Hank & Oppenheimer s.n. | BISH | Molokai, Hawaii | KF688452 | KF688367 | KF688284 | - |
| *C. virescens* | A. Markey & J. Cantley NZ239 | HAW | Otago Penninsula, New Zealand | KF688421 | KF688338 | KF688254 | KT861951 |
| *C. waima* | J. Cantley 196 | HAW | cult. Auckland, New Zealand | KF688468 | KF688383 | KF688300 | KT861991 |
| *C. waimeae* | J. Cantley 54 | HAW | Kauai, Hawaii | KF688466 | KF688381 | KF688298 | KT861989 |
| *C. wallii* | J. Cantley 212 | HAW | Wellington, New Zealand | KF688422 | KF688339 | KF688255 | KT861952 |
| *D. paludosa* | NSW 154507 | NSW | Australia | KF688423 | - | AF257917.1 | - |
| *L. setulosum* | J. Cantley 208 | HAW | Auckland Botanical Garden | KT861897 | - | KT861919 | KT862015 |
| *N. depressaa* | A. Markey & J. Cantley NZ230 | HAW | Dunedin, New Zealand | KF688471 | KF688386 | KF688303.1 | - |
| *N. dichondrifolia* | A. Markey | CHR | New Zealand | KF688472 | KF688387 | KF688304 | - |
| *N. granadensis* | J. Cantley 38 | HAW | Oahu, Hawaii | KF688451 | KF688366 | KF688283 | - |
| *No. neocaledonica* | Munzinger & McPherson 532 | MO | New Caledonia | KF688454 | KF688369 | KF688286 | KT861978 |
| *O. vaginata* | A. Markey 6306 | PERTH | Australia: Western Australia: woodvale wildlife reserve | AF257935.1 | - | KT861920 | KT862013 |
