# Supplementary material for: Biogeography and evolutionary diversification in one of the most widely distributed and species rich genera of the Pacific
Source: AoB Plants. 2016 Aug 2;8:plw043. doi: 10.1093/aobpla/plw043 (PMC4972462; doi:10.1093/aobpla/plw043)
Supplement: Supplementary Data [file supp_plw043_suppl_data.zip › aobplants-15339-s02.docx]

Dear J. Hall Cushman et al,

Thank you for the email and alerting me to the choice for this MS to be featured as “Editor’s Choice.” Thank you for your patience as I’m still transitioning from the Australian time change in the field. Below I have prepared answers to the questions from your email. My responses are in red type face.

Best,

Jason T. Cantley

 1)   Topics selected from our subject list that best categorize your paper (please let us know if you think a topic needs to be added to our list). The topic list is accessed using the following link: 

<[http://www.oxfordjournals.org/our_journals/aobpla/topiclist.html](http://www.oxfordjournals.org/our_journals/aobpla/topiclist.html)>

Phylogeny & Systematics

2) An 80-100-word promotional statement that summarizes the results of your paper in a compelling way and is written in a relatively jargon-free style that is suitable for a general scientific audience.

The largest natural feature on Earth is the Pacific Ocean, which covers over one-third of our planet’s surface. This study reconstructed the previously unknown historical biogeography of *Coprosma* (Rubiaceae), which is one of the largest (>110 species) and most widespread flowering plant genera distributed across the Pacific. A New Zealand origin of *Coprosma* was inferred at approximately 25 million years ago (Ma), but most of the distribution was achieved 6 Ma likely by frugivorous birds. Over 30 dispersals events are inferred and >8 locations were colonized more than once, which is perhaps more than any Pacific-centered genus investigated to date.

3) A high-quality photograph or other image (preferably in colour) relevant to the manuscript (it may be one already included in the paper or a new image), with a brief explanatory caption that includes acknowledgement of the image source. Please aim for an image resolution of about 300 dpi at 10 x 10 cm and simply paste it into your cover letter. Permission from the copyright holder will be required for any images that are not original, including images from the Internet, unless they are considered to be in the public domain. 

**Note: High-resolution photos are also included as separate files and imbedded here. Permission was granted from Maggie J. Sporck-Koehler.

Filename: Clongifolia_WaianaeCliff.jpg

**
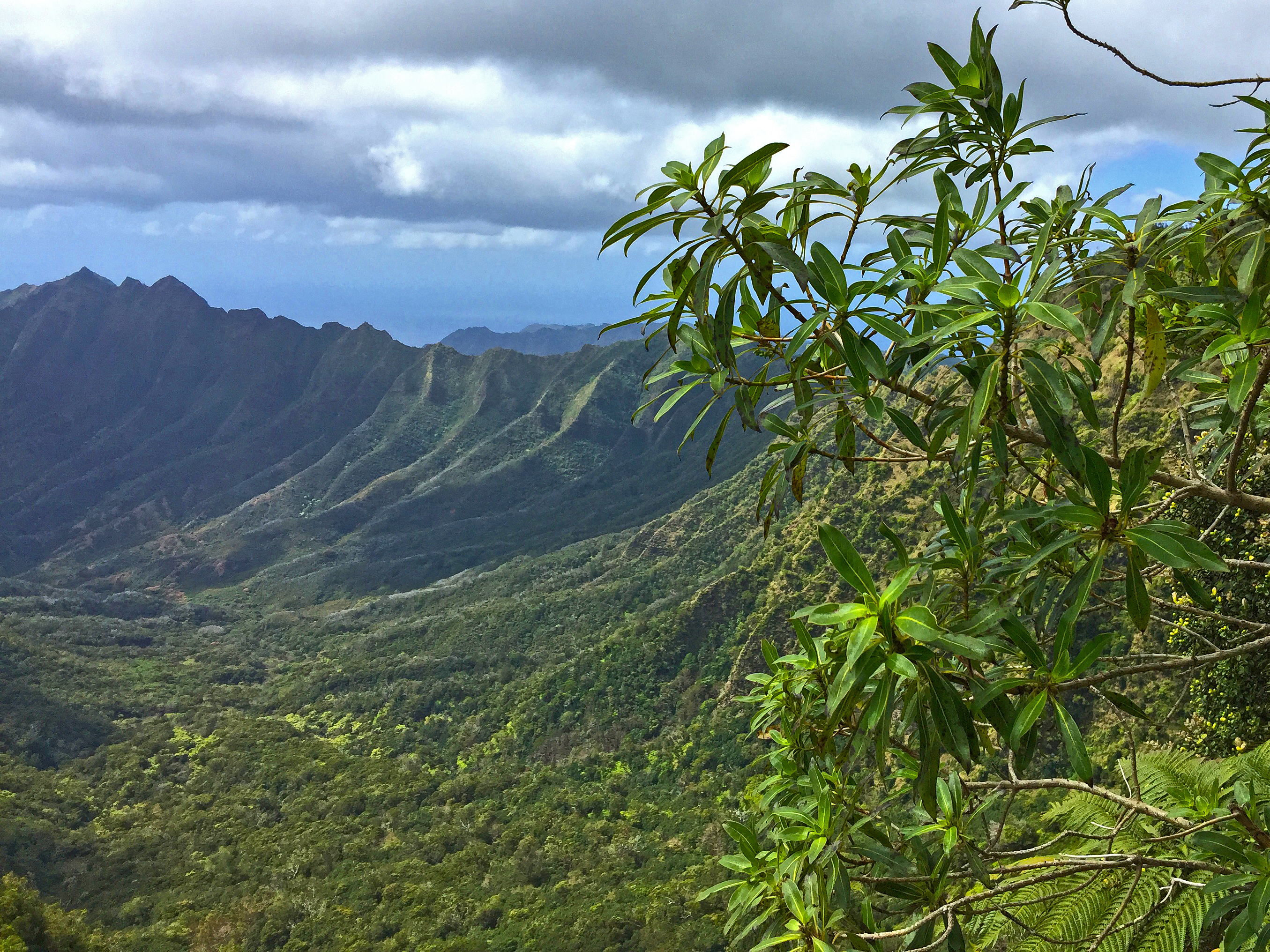
**

Figure Caption: Typical island cliff habitat of *Coprosma longifolia* overlooking Waianae Kai Valley, O‘ahu of the Hawaiian Islands. Photo credit: Maggie J. Sporck-Koehler.

Filename: Cfoliosa_Waikolu.jpg

**
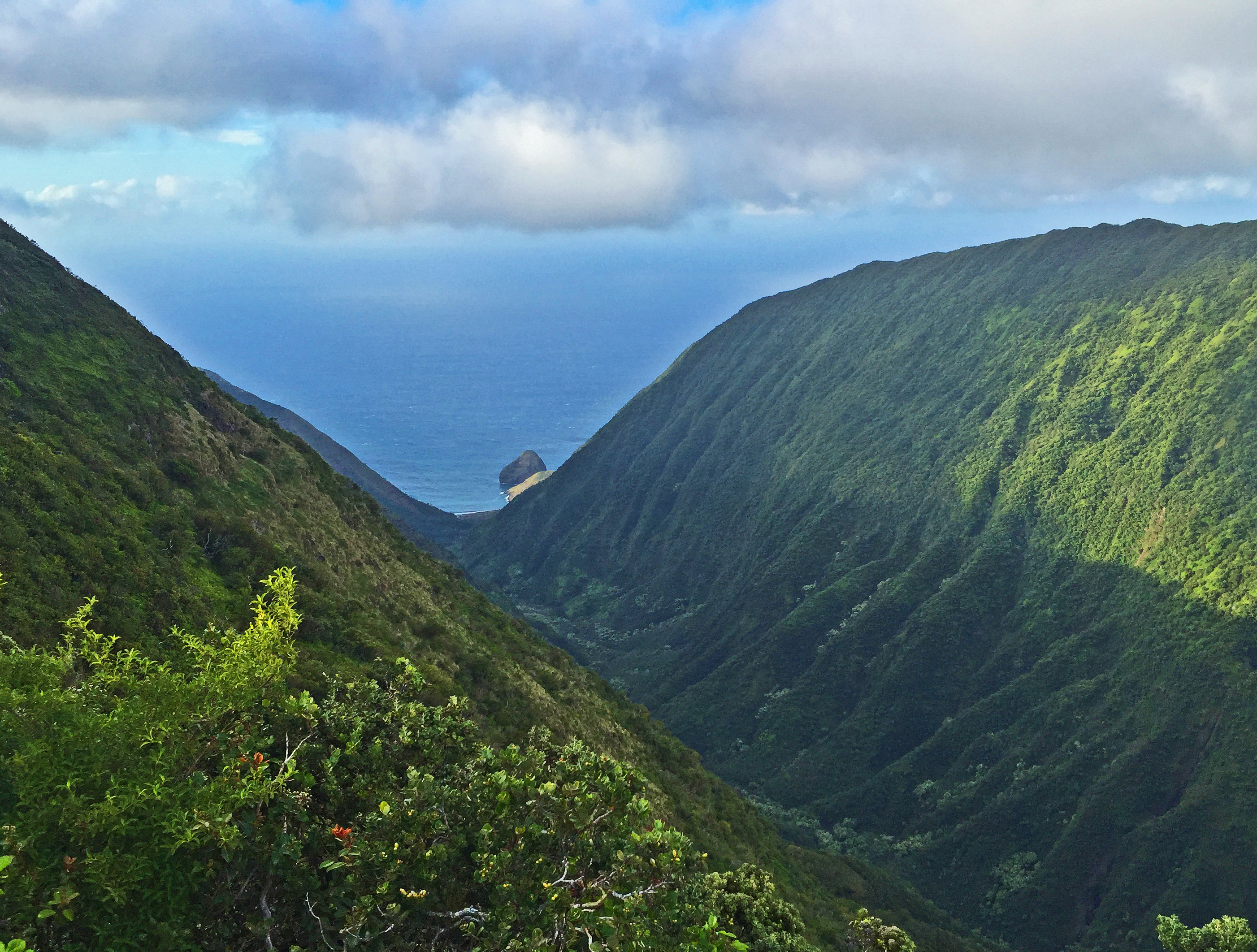
**

Figure Caption: The Hawaiian species *Coprosma foliosa* (light yellow-green leaves, bottom left) on the cliff face of Waikolu Valley, Moloka‘i looking north towards the Pacific Ocean. Photo credit: Jason T. Cantley.

4) A list of up to 10 email addresses of colleagues to whom you would like us send a PDF link to your paper once it published fully-formatted.

[cmorden@hawaii.edu](mailto:cmorden@hawaii.edu)

[ranker@hawaii.edu](mailto:ranker@hawaii.edu)

[lorenece@ntbg.org](mailto:lorenece@ntbg.org)

[wagner@si.edu](mailto:wagner@si.edu)

[funkv@si.edu](mailto:funkv@si.edu)

[mmchau@hawaii.edu](mailto:mmchau@hawaii.edu)

[Margaret.J.Sporck-Koehler@hawaii.gov](mailto:Margaret.J.Sporck-Koehler@hawaii.gov)

[swalsh@ntbg.org](mailto:swalsh@ntbg.org)

[Michael.kiehn@univie.ac.at](mailto:Michael.kiehn@univie.ac.at)

[kentaw@hawaii.edu](mailto:kentaw@hawaii.edu)

5) The name of any Special Issue for which your paper is being prepared. 

Celebrating Carlquist's Legacy

Items 2, 3 and 4 will be used to promote your paper on-line. Any image that is neither original nor in the public domain will require permission from the copyright holder.
