# Supplementary material for: Biogeography and evolutionary diversification in one of the most widely distributed and species rich genera of the Pacific
Source: AoB Plants. 2016 Aug 2;8:plw043. doi: 10.1093/aobpla/plw043 (PMC4972462; doi:10.1093/aobpla/plw043)
Supplement: Supplementary Data [file supp_plw043_suppl_data.zip › aobplants-15339-s03.doc]

# OPEN ACCESS – RESEARCH ARTICLE

# Biogeography and evolutionary diversification in one of the most widely distributed and species rich genera of the Pacific

### Jason T. Cantley^1,2,^*, Adrienne S. Markey^3,4^, Nathan G. Swenson^5^ and Sterling C. Keeley^1^

^1^Department of Botany, University of Hawai‘i at Mānoa, Honolulu, Hawai‘i 96822, USA.

^2^Department of Biology, Bucknell University, Lewisburg, Pennsylvania 17837, USA.

^3^Department of Botany, University of Otago, Dunedin 9016, New Zealand.

^4^Department of Parks and Wildlife, Science and Conservation Division, Kensington, Western Australia 6151, Australia.

^5^Department of Biology, University of Maryland, College Park, Maryland 20742, USA.

*Corresponding author

Corresponding author’s e-mail address: jason.cantley@bucknell.edu

Running head: Pacific biogeography of *Coprosma* (Rubiaceae)
